# Homology-Directed Repair in Zebrafish: Witchcraft and Wizardry?

**DOI:** 10.3389/fmolb.2020.595474

**Published:** 2020-12-07

**Authors:** Kendal Prill, John F. Dawson

**Affiliations:** Department of Molecular and Cellular Biology, University of Guelph, Guelph, ON, Canada

**Keywords:** CRISPR, genome modification, homology directed repair, zebrafish, precision medicine and genomics

## Abstract

Introducing desired mutations into the genome of model organisms is a priority for all research focusing on protein function and disease modeling. The need to create stable mutant lines has resulted in the rapid advancement of genetic techniques over the last few decades from chemical mutagenesis and zinc finger nucleases to clustered regularly interspaced short palindromic repeats (CRISPR) and homology-directed repair (HDR). However, achieving consistently high success rates for direct mutagenesis in zebrafish remains one of the most sought-after techniques in the field. Several genes have been modified using HDR in zebrafish, but published success rates range widely, suggesting that an optimal protocol is required. In this review, we compare target genes, techniques, and protocols from 50 genes that were successfully modified in zebrafish using HDR to find the statistically best variables for efficient HDR rates.

## Introduction

Introducing specific mutations into the genome of model organisms is a long-standing goal for many researchers. With the rapid development of genome-editing tools, targeted editing is becoming achievable. Since 2013, there have been hundreds of genes in zebrafish that have been successfully modified using homology-directed repair^1^ (HDR; Table 1). However, the success rate for modifying the genome is inconsistent due to a variety of protocols. For the purposes of this review, success of HDR is defined as the permanent repair/integration of the single-nucleotide polymorphism (SNP)/exogenous DNA. Imperfect HDR introduces indels into genes that likely interfere with expressed protein function and will not be discussed in this review.

**TABLE 1:** Successfully modified genes using homology-directed repair (HDR).

**Gene**	**Reference**	**Gene**	**Reference**	**Gene**	**Reference**
*tyrosinase (tyr)*	Hisano et al., 2015; Moreno-Mateos et al., 2017; Bai et al., 2020	*nefma*	Eschstruth et al., 2020	*flna*	Wierson et al., 2020
*tbx20*	Burg et al., 2018	*th*	Bai et al., 2020	*noto*	Wierson et al., 2020
*krtt1c19e*	Hisano et al., 2015	*fh*	Hwang et al., 2013	*s1pr1*	Wierson et al., 2020
*twist2*	Bai et al., 2020	*etv2*	Chang et al., 2013	*rb1*	Wierson et al., 2020
*rpl18*	Bai et al., 2020	*smad6a*	Boel et al., 2018	*msna*	Wierson et al., 2020
*aldh1a2*	Burg et al., 2018	*ntla*	Moreno-Mateos et al., 2017	*cx43.4*	Wierson et al., 2020
*ybx1*	Zhang et al., 2018	*pln*	Tessadori et al., 2018	*anxa2a*	Wierson et al., 2020
*fabp10a*	DiNapoli et al., 2020	*gskba*	Hwang et al., 2013	*kdrl*	Wierson et al., 2020
*albino*	Irion et al., 2014; Moreno-Mateos et al., 2017	*tyrp1b*	DiNapoli et al., 2020	*vegfaa*	Wierson et al., 2020
*gata1a*	Luo et al., 2018	*fleer*	Burg et al., 2018	*esama*	Wierson et al., 2020
*nop56*	Bai et al., 2020	*golden*	Moreno-Mateos et al., 2017; DiNapoli et al., 2020	*aqp8a1*	Wierson et al., 2020
*fli1a*	Luo et al., 2018	*pbx4*	Farr et al., 2018	*aqp1a1*	Wierson et al., 2020
*abcc9*	Tessadori et al., 2018	*tprkb*	Boel et al., 2018	*tbx18*	Burg et al., 2016
*mitfa*	DiNapoli et al., 2020	*tardbp*	Armstrong et al., 2016	*h3f3a*	DiNapoli et al., 2020
*rps14*	Bai et al., 2020	*fus*	Armstrong et al., 2016	*kcnj8*	Tessadori et al., 2018
*gfap*	Luo et al., 2018	*slc2a10*	Boel et al., 2018	*lcp1*	Baumgartner et al., 2019
*tcf21*	Burg et al., 2018	*pls3*	Boel et al., 2018		

This review will discuss the HDR protocols currently used for the modification of the zebrafish genome. The majority of zebrafish studies agree: only sgRNAs with high cutting efficiencies (>60%) should be used, chemical modification of the repair template was not present, the repair template must overlap the double-strand break (DSB) site (asymmetrically or symmetrically), microinjections occur during the 1–2 cell stage, the protospacer adjacent motif (PAM) site must be altered to prevent cutting of successfully repaired targets, and the DSB cut site should be within 20 nucleotides of the target nucleotide (Paquet et al., 2016; Boel et al., 2018; Burg et al., 2018; Bai et al., 2020). There are few exceptions to the target/cut site proximity standard where cut sites are a significant distance away from their target loci (Moreno-Mateos et al., 2017; Luo et al., 2018; Eschstruth et al., 2020). These steps are the standard practice in the field, so the range of HDR success rates mainly stems from variations of those steps.

The variations in HDR protocols include the type of template, length of the homology arms, symmetry of repair template, choice of endonuclease, endonuclease mRNA or protein, targeted strand, injection into the yolk or cell, the introduction of non-homologous end joining (NHEJ)-inhibiting or HDR-enhancing drugs, injection or incubation with inhibitors. With all these variations, we asked: Which factors contribute to the highest success rate?

To answer this question, we analyzed the conditions used to successfully modify 50 genes in zebrafish using HDR to identify statistically advantageous specifications for a targeted modification protocol. Some of these genes have been modified across multiple publications providing direct comparison of different HDR approaches. The techniques and target genes discussed in this review met five key criteria for evaluation: (1) the technique must use clustered regularly interspaced short palindromic repeats (CRISPR) for modification; (2) an endonuclease (e.g., Cas9, Cpf1, etc.) must be used for DNA cutting; (3) the study must focus on genome modification using HDR; (4) a rate of HDR in either somatic tissue or germline transmission is reported; and (5) at minimum, a brief description of the repair template and protocol is provided. This review focuses on studies that had a shared focus for determining the best conditions for improving HDR; therefore, we could not evaluate methods used in other research where the criteria for evaluating were not presented. Overall, we found that DNA topology is an important factor, and we describe best practices for protocols with high rates of HDR.

## Overview of Homology-Directed Repair Mechanisms

Double-strand breaks in DNA can occur for many reasons: normal cellular functions such as metabolic by-products and management of DNA during mitosis and meiosis. Other sources of DNA damage come from external sources such as radiation, drugs, or *in vitro*-derived endonucleases (e.g., Cas9) (Negritto, 2010). There are two major methods utilized by cells for DSB repair: NHEJ and homologous recombination (HR) (Chapman et al., 2012; Marini et al., 2019). NHEJ is an error-prone process because broken ends of DNA are directly ligated together regardless of sequence homology or damage (Rulten and Grundy, 2017; Pannunzio et al., 2018). The error-prone NHEJ repair pathway is often exploited by researchers to generate knock-out mutants as there is no need to produce a specific nucleotide sequence in the target gene. HR is not error-proof but most often results in seamless DNA repairs because the cell machinery uses homologous sequences to ligate broken ends together or fill deleted sequences that then preserve the original genomic sequence (Ranjha et al., 2018; Seol et al., 2018; Wright et al., 2018). HDR has emerged in recent years as a way to generate animals with specific nucleotide sequences to study protein function or model disease(s). HR is a broad reference to several mechanisms for DNA repair that uses a template. Microhomology-mediated end-joining (MMEJ), single-strand annealing (SSA), DSB repair (DSBR), synthesis-dependent strand annealing (SDSA), and break-induced replication (BIR) were reviewed in Krogh and Symington (2004), McVey and Lee (2008), Huertas (2010), Chang et al. (2017), and Ranjha et al. (2018).

After a DSB is introduced into the genome, proteins are recruited to the exposed DNA ends to stabilize the break and initiate repair (Liu and Huang, 2016). DNA end resection, which typically occurs immediately after the DSB, is one of the major deciding factors between a cell using NHEJ or HR mechanisms for DNA repair (McVey and Lee, 2008; Huertas, 2010; Symington and Gautier, 2011; Chang et al., 2016; Marini et al., 2019). End resection results in a 5′→ 3′ digestion of DNA ends to create single-stranded overhangs that are conducive to HR (Liu and Huang, 2016) – a cellular decision/process that can be manipulated by researchers to encourage HR instead of NHEJ for genomic modification.

There are several proteins involved in DNA resection, NHEJ, and the umbrella of HR mechanisms. While some of these proteins are targets of HDR methods to enhance precise modification (Sanford-Crane et al., 2018), a detailed account of all proteins involved is beyond the scope of this review. Extensive reviews of NHEJ and HR pathways are reviewed by Rulten and Grundy (2017) and Sun et al. (2020), respectively.

## Trends for Genes With Successful Homology-Directed Repair

### DNA Topology

**FIGURE 1:**
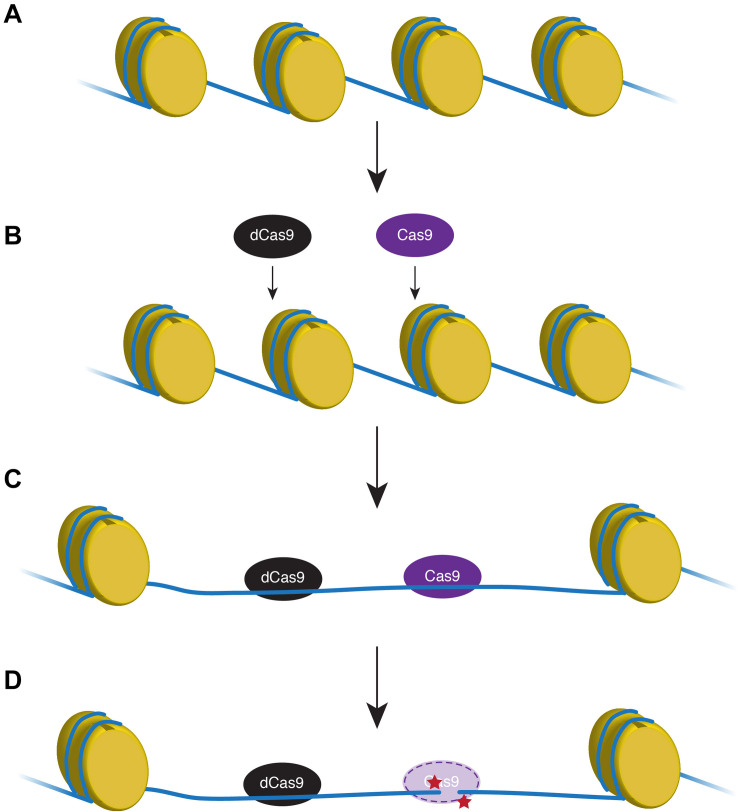
Proxy-clustered regularly interspaced short palindromic repeats (CRISPR) technique expands the region of relaxed DNA. In transcriptionally inactive regions, DNA (blue line) is tightly wound around histones (yellow) that inhibits endonuclease access to the DNA **(A,B)**. A catalytically non-functional Cas9 (dCas9; black) removes nucleosomes and opens a wider region of DNA proximal to the target loci that will be modified using active Cas9 (purple; **C**). Active Cas9 cuts the DNA to create a double-strand break (red stars) that can be manipulated for homology-directed repair **(D)**.

The genomic landscape and properties of 50 targeted zebrafish genes were analyzed. Several recent studies have shown that target gene cutting by an endonuclease is directly inhibited by the presence of nucleosomes (Horlbeck et al., 2016; Yarrington et al., 2018; Liu et al., 2019), although a study from 2015 suggests zebrafish do not adhere to this chromatin restriction (Moreno-Mateos et al., 2015). Moreno-Mateos et al. (2017) found using a catalytically inactive form of Cas9 to unwind DNA near the HDR target locus significantly increased the efficiency of active endonuclease (Figure 1).

We hypothesized that the DNA topology impacts the success rate of HDR when the DNA is open during the time that Cas9 ribonucleo–protein complex is active. Injected Cas9 ribonucleo–protein complex has been shown to remain to the 90% epiboly (9 hpf) stage (Burger et al., 2016); however, Cas9 protein synthesized from injected Cas9 mRNA may be present at later stages due to a delay in Cas9 protein production from the injected mRNA. However, in order to achieve successful HDR germline transmission and establishment of a desired zebrafish line, permanent genomic modifications must occur before specification of the primordial germ cells (PGCs). By 4 hpf, zebrafish primordial germ cells are specified and the somatic gene program is transcriptionally inactivated, making the accessibility of target genes more challenging after 4 hpf (Köprunner et al., 2001; Wolke et al., 2002; Gross-Thebing et al., 2017). However, we would like to highlight that there are limitations to the 4-hpf topology approach, unique to every gene, due to possible interference from maternal mRNA and protein contributions (Tadros and Lipshitz, 2009; Langley et al., 2014).

**FIGURE 2:**
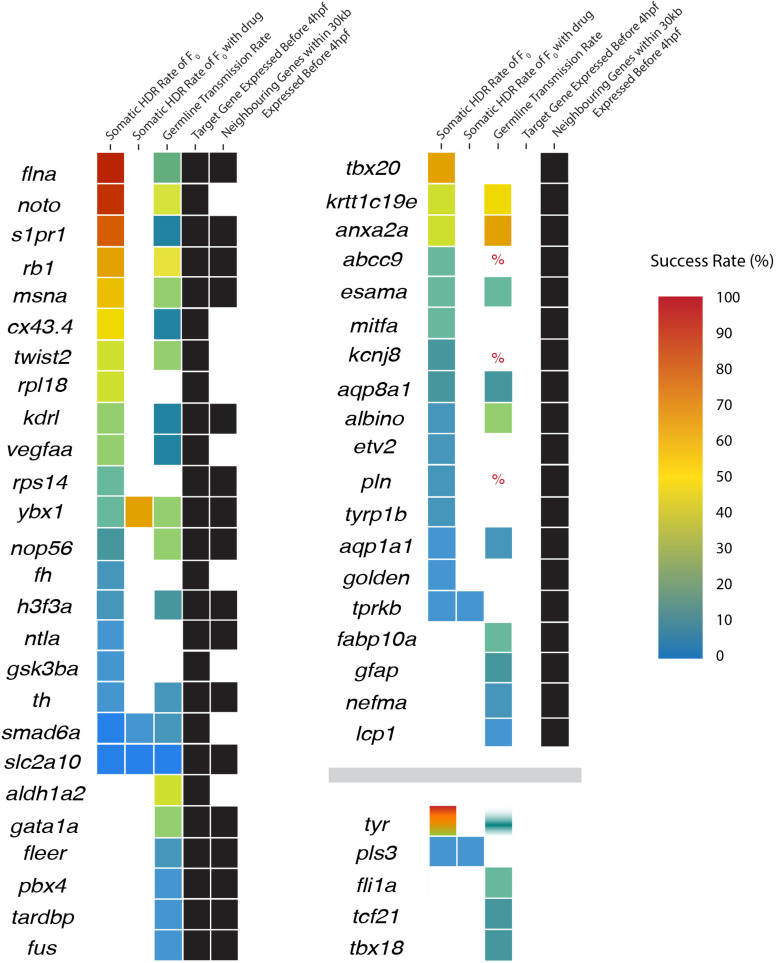
Expression status of neighboring genes and the success rates of homology-directed repair (HDR) in target genes. Genes are initially sorted into three categories according to target and neighboring gene expression (black boxes): (1) target genes expressed before 4 hpf (regardless of neighboring gene expression), (2) target gene is not expressed but neighboring genes are expressed before 4 hpf, and (3) the target and neighboring genes are not expressed before 4 hpf. Within each category, genes are sorted in descending somatic HDR rates followed by descending germline transmission rates when no somatic rate was provided. HDR rates are displayed as a heat map with color-coded success rates for both somatic and germline transmission rates (percent sign indicates a study where success was mentioned but no rate was provided or could be determined; white/blank boxes indicate no rate). *Tyrosinase* includes four colors from four independent studies, with only one study providing a germline transmission rate.

To test the hypothesis that DNA topology impacts the success rate of HDR, we compared the timing of target zebrafish gene expression onset (Ruzicka et al., 2019; Papatheodorou et al., 2020) with the reported Cas9 activity and before PGCs are specified at 4 hpf. The 50 genes examined were sorted based on expression before or after 4 hpf (Figure 2 and Table 2 and Supplementary Figure 1).

**TABLE 2:** Genes expressed before primordial germ cells (PGC) specification and during the Cas9 active period.

	**PGC specification**		**Cas9 protein**	
	**Before 4 hpf**	**After 4 hpf**	**Before 9 hpf**	**After 9 hpf**
Number of Genes	26	24	33	17
*T*-test	0.69		0.001	

*Onset of gene expression was determined using the collated research provided on zfin.org and EMBL-EBI Expression Atlas for zebrafish. Genes were sorted into groups based on expression before or after 4 or 9 hpf. A two-tailed *T*-test with unequal variance was performed to determine the significance between gene expression onset time and success of homology-directed repair (HDR).*

Twenty-six of the 50 target genes examined were expressed before 4 hpf (Ruzicka et al., 2019; Papatheodorou et al., 2020). We found a significant difference in the success of somatic HDR when a gene was expressed during the active period for Cas9 endonuclease activity, supporting a model that the state of the target gene DNA affects the ability to modify that gene (Table 2). For a number of genes/studies examined, the germline transmission rate was similar or higher than the somatic HDR rate (e.g., *rb1*, *msna*, *twist2*, *krtt1c19e*, *anxa2a*, *esama*, *ybx1*, *aqp8a1*, *nop56*, *albino*, *h3f3a*, *th*, *smad6a*, *aqp1a1*, and *slc2a10*; Figure 2), suggesting that unknown germline rates may be equally successful to the somatic rates reported by some labs (outliers with high somatic HDR/low germline transmission rate originated from one paper: *flna*, *1 tyr study*, *noto*, *s1pr1*, *cx43.3*, *kdrl*, and *vegfaa*; Figure 2).

This model might suggest successful HDR is limited only to genes of interest expressed before 4 hpf; however, the pool of successfully modified genes contained a number of genes expressed later in zebrafish development. Since the topology of a target gene may be impacted by the state of the surrounding DNA, we expanded our analysis to genes proximal to the target genes (Figure 2 and Supplementary Figure 1).

The 24 target genes expressed after 4 hpf were sorted based on whether the expression of surrounding genes occurs before 4 hpf (Ruzicka et al., 2019; Papatheodorou et al., 2020). By limiting the analysis to 30 kb upstream and downstream of the target gene, 19 of the 24 target genes had neighboring genes expressed before 4 hpf (Figure 2 and Supplementary Figure 1). Taken together, 45 out of the 50 genes reviewed here have relaxed DNA in the target genes or their neighbors when Cas9 is active and before PGCs are specified, based on our current gene expression information.

While we see a pattern with DNA topology, there is clearly more to HDR success rates since *tyr*, one of the most successful somatically modified genes reviewed here is expressed after Cas9’s 9 hour active period and does not have any proximal (within 30 kb) neighboring genes expressed before 9 hpf. Cases such as *tyr* suggest that other factors contribute to the overall HDR success rate, such as variations in the techniques and protocols as reviewed in the next section.

## Different Techniques and Protocols for Successful Homology-Directed Repair

We compared the techniques and protocols that lead to successful modification of the 50 zebrafish genes reviewed here to determine if there is a common best practice.

### Cell vs. Yolk

The majority of HDR research reports injecting reagents into the cell cytoplasm of 1- or 2-cell stage embryos with germline success rates ranging from 0 to 63.9% (Wierson et al., 2020). Germline transmission success rates from injecting into the yolk range from 12.5% (Burg et al., 2016) to 37.5% (Burg et al., 2018), although this range results from a limited number of studies. Comparison of the most successfully modified genes (somatic and germline modified), *flna*, *tyr*, *noto*, *tbx20*, *rb1*, *msna*, *twist2*, *rpl18*, *krtt1c19e*, *anxa2a*, and *ybx1* (with use of drug), suggests there is an advantage to injecting into the cell vs. the yolk (Hisano et al., 2015; Moreno-Mateos et al., 2017; Burg et al., 2018; Zhang et al., 2018; Bai et al., 2020; Wierson et al., 2020). Comparing the techniques used for these 11 genes and 14 modifications (*tyr* was modified by four different labs), 12 out of 14 techniques utilized injecting into the cytoplasm of the 1- or 2-cell stage embryo. Therefore, we suggest that injecting into the cell cytoplasm yields the best HDR rates with respect to both somatic tissue and germline transmission.

### Injection Mixture

The concentrations of injection mixture reagents varied significantly across the studies reviewed here. Based on the protocols provided, we converted all Cas9 mRNA or protein, sgRNA, and template DNA to picomoles injected and then determined a ratio of these reagents for each protocol (Table 3). Most of the successful protocols used an injection mixture ratio where the concentration of Cas9 mRNA/protein was at least four times the concentration of sgRNA and six times the concentration of template injected. There was no significant trend in the ratio of sgRNA to template injected, suggesting the optimal injection mixture depends on the amount of endonuclease injected.

**TABLE 3:** Injection mixture ratios from the most successful homology-directed repair (HDR) protocols.

**Target gene(s)**	**Reference**	**Somatic HDR success rate (%)**	**Ratio: Cas9 mRNA/protein: sgRNA: template**
*flna, tyr, noto, s1pr1, msna, cx43.4, anxa2a, kdrl*	Wierson et al., 2020	100, 63.6, 95, 80.9, 55.1, 50, 35.3, 34.8	15:2.5:1
*rb1*	Wierson et al., 2020	60.1	30:2.5:1
*tyr, twist2*	Bai et al., 2020	98.5, 40	30:3:1
*tbx20, aldh1a2, tcf21, fleer*	Burg et al., 2018	68.8, 37.5, 16.7, 4.8	1:1.6:555.56
*ybx1*	Zhang et al., 2018	25	4:1:4
*tyr, krtt1c19e*	Hisano et al., 2015	77	10:1:1
*albino*	Irion et al., 2014	24	50:3:1
*gata1a, fli1a, gfap*	Luo et al., 2018	23.6, 21.8, 17	6:1:1
*abcc9, kcnj8, pln*	Tessadori et al., 2018	21.4, 15.4, 6.7	6:1:1
*nefma*	Eschstruth et al., 2020	10	8:5:1

*All HDR reagent concentrations or masses were converted to picomole injected for each study. Picomole values were used to determine the ratio of endonuclease, sgRNA and template with 1 representing the reagent with the smallest mass injected per protocol.*

#### Endonuclease mRNA **v**s. Protein

The use of Cas9 mRNA or Cas9 protein varies within the field. The highest success rates for germline transmission of HDR modifications utilized Cas9 mRNA and long single-stranded oligonucleotides or double-stranded templates (Bai et al., 2020; Wierson et al., 2020); however, no comparisons with Cas9 protein have been made (Hisano et al., 2015). A 2018 study compared HDR success using Cas9 mRNA vs. Cas9 protein on the *ybx1* gene (Zhang et al., 2018). When using a plasmid or a double-stranded DNA (dsDNA) template as the donor, the HDR success rates of Cas9 mRNA or protein were similar (16 vs. 15%, 8 vs. 10%, respectively). Interestingly, the use of Cas9 protein made a significant difference in HDR success rate when using an ssODN as a donor template when compared to Cas9 mRNA (0 vs. 5%, respectively) (Zhang et al., 2018).

The highest HDR success rates (somatic and/or germline, Figure 2) from the studies reviewed here used endonuclease mRNA (Hisano et al., 2015; Moreno-Mateos et al., 2017; Burg et al., 2018; Bai et al., 2020). Using Cas9 protein has been shown to significantly improve germline transmission rates of modified genes over Cas9 mRNA (33.3 vs. 13.8%, respectively) when somatic rates were similar (Zhang et al., 2018). Therefore, it would be interesting to compare the results of HDR germline transmission rates of the most successfully modified genes employing Cas9 protein. Until such an investigation with Cas9 protein is performed, the current information suggests that Cas9 mRNA is the best option for HDR in zebrafish.

#### Template Format and Design Elements

The choice of repair template remains one of the key components researchers manipulate for successful HDR. Currently, plasmids, dsDNA, single-stranded oligonucleotide (ssODN; ∼60–120 nt), and long single-stranded DNA (lssDNA; ∼300–700 nt) are used as repair templates for HDR (Hisano et al., 2015; Moreno-Mateos et al., 2017; Boel et al., 2018; Burg et al., 2018; Bai et al., 2020; Wierson et al., 2020). The genes with the highest HDR success rates (Figure 2) utilized single-stranded repair templates (Moreno-Mateos et al., 2017; Burg et al., 2018; Bai et al., 2020) or double-stranded templates, although the use of double-stranded templates resulted in both the highest (*anxa2a*: 63.9%) and lowest (*s1pr1, cx43.4, kdrl, vegfaa*: 0%) germline transmission rates (Wierson et al., 2020).

Of note are two contrasting studies comparing HDR rates of ssODNs and plasmids. Zhang et al. (2018) showed that plasmid repair templates yielded significantly higher *ybx1* gene HDR success than ssODN templates. In contrast, Bai et al. (2020) showed that ssDNA templates were superior to plasmids or linear dsDNA for HDR. Furthermore, lssDNA templates produced higher HDR rates than ssODN templates for every gene tested (Bai et al., 2020). These data suggest that single-stranded templates are the best option for somatic and germline genome modification in zebrafish. In the next section, we examine the properties of the most successful templates.

##### Homologous arm size and symmetry

Homologous arm size significantly affects repair rates for a gene when all other conditions are constant (Hisano et al., 2015; Bai et al., 2020). Increasing the length of the long single-stranded template from 300 to 500 nucleotides decreased HDR efficiency by 10%; this efficiency drops by 59% when using shorter ssODN (Bai et al., 2020). Other genes repaired with 300 nucleotide lssDNA templates demonstrated an impressive rate of HDR (11–40%) over other template sizes, although these rates were lower than the 98.5% achieved while modifying *tyr* (Bai et al., 2020).

Targeting *tyr* for modification with a donor plasmid, Hisano et al. (2015) demonstrated that 40 bp symmetrical homologous arms resulted in a 77% success rate, with the rate dropping as the homologous arms were decreased to 10 bp (60%). Bai et al. (2020) also modified *tyr* with a donor plasmid template containing symmetrical ∼740 bp homologous arms but only achieved a rate of 5.4%. Wierson et al. (2020) performed a detailed comparison of homology arm length and showed that short homology arms (24 and 48 bp) resulted in efficient integration of donor templates when compared to long homology arms (1 kb). The use of symmetrically homologous 400 bp arms to modify *ybx1* with a donor plasmid resulted in an HDR rate of 55–58% (Zhang et al., 2018), suggesting that homologous arm length with plasmid templates can greatly affect HDR.

Burg et al. (2018) showed that the asymmetrical homologous arms of a 120-nucleotide ssODN repair template, with either arm no smaller than 18 nucleotides, achieved a somatic HDR rate of 68.8%. Conversely, a ∼107-nucleotide ssODN with symmetrical 50-nucleotide homologous arms resulted in a somatic HDR rate ranging from 0 to 5% when targeting *ybx1* (Zhang et al., 2018). Boel et al. (2018) analyzed the success of HDR with ssODN templates with four genes, examining the impact of strand complementary, asymmetrical and symmetrical homologous arms, and homologous arm length between 30 and 90 nucleotides. The HDR rates from this study ranged from 1.2 to 7.2% (Boel et al., 2018). Wierson et al. (2020) used short symmetrical homologous arms for the majority of their precise modifications, but this study produced the highest and lowest germline HDR rates (0–63.9%). This range of HDR rates may be due to the combination and positioning of the homologous arms with respect to the sgRNA(s), the endonuclease used, and other cellular machinery that is unique to each gene. Currently, there is no clear design to a single-stranded DNA template with higher HDR rates. However, it seems that homologous arms that are too large (>400 nucleotides) and too short (<18 nucleotides) are inefficient for the purpose of HDR and establishment of precisely modified zebrafish lines. Comparison of all studies reviewed here demonstrated no significant difference between the use of symmetrical vs. asymmetrical homology arms.

One model based on our analysis is that the range of successful arm size and symmetry of repair templates is partly due to the landscape at the target locus, especially for those target genes that are not expressed during the active period for Cas9. While Cas9 endonucleases can relax DNA and remove nucleosomes to a degree, long repair templates or homologous arms may exceed the Cas9-relaxed region, reducing the success of HDR. Approaches such as proxy-CRISPR should be investigated further to increase the success of modifying the zebrafish genome (Chen et al., 2017).

#### Small Interfering Molecules–Drugs

Small interfering molecules inhibiting specific factors of NHEJ in combination with HDR reagents are thought to favor HDR for DNA repair (Boel et al., 2018; Zhang et al., 2018; Aksoy et al., 2019). However, the number of studies examining the effect of drugs on the efficiency of HDR in zebrafish is small. There is variability in the success rates of HDR after drug treatments, and only five drugs have been tested in zebrafish, with three commonly used across three studies.

The small interfering molecule drugs used with zebrafish HDR work can be separated into two categories: (1) inhibitors of NHEJ machinery and (2) enhancers of HDR (Table 4). SCR7, a DNA Ligase IV inhibitor, and Nu7441 and KU0060648, inhibitors of DNA-PK, are used to inhibit cell machinery of the NHEJ pathway. Enhancers of the HDR pathway are RS-1, a RAD51 stimulant, and L755507, a β3-adrenergic receptor agonist. The three most commonly used drugs are SCR7, Nu7441, and RS-1 that each target a different protein involved in DNA repair.

**TABLE 4:** Small interfering molecules tested in zebrafish to improve homology-directed repair (HDR) rates.

**Drug**	**Target cell machinery**	**Process affected**	**Proposed result**	**Study tested**
SCR7	DNA Ligase IV	NHEJ	Inhibits NHEJ pathway–promotes other repair mechanisms	Boel et al., 2018; Zhang et al., 2018; Aksoy et al., 2019; Bai et al., 2020
Nu7441	DNA-PK	NHEJ	Inhibits NHEJ pathway–promotes other repair mechanisms	Boel et al., 2018; Zhang et al., 2018; Aksoy et al., 2019
KU0060648	DNA-PK	NHEJ	Inhibits NHEJ pathway–promotes other repair mechanisms	Boel et al., 2018
RS-1	RAD51	DNA Strand Exchange	Stabilizes RAD51–enhances strand exchange	Boel et al., 2018; Zhang et al., 2018; Aksoy et al., 2019
L755507	β3-adrenergic receptor	DNA Repair	Stimulates β3-adrenergic receptor–activates DNA repair pathways	Boel et al., 2018

*NHEJ, non-homologous end joining.*

SCR7 was beneficial for the genetic modification of *ybx1* (Figure 2 and Supplementary Figure 1; Zhang et al., 2018) but not in the homologous repair of *smad6*, *tprkb*, *pls3*, *slc2a10*, *tyr*, *twist2*, *nop56*, *rpl18*, *rps14*, and the *acta1* promoter (Boel et al., 2018; Aksoy et al., 2019; Bai et al., 2020). SCR7 significantly increased HDR when using ssDNA (5–13%), dsDNA (10–25%), and plasmid (16–58%) repair templates targeting *ybx1* (Zhang et al., 2018).

The use of RS-1, an HDR enhancer, provided a small increase in HDR success rates when compared to SCR7 (Boel et al., 2018; Zhang et al., 2018; Aksoy et al., 2019). When RS-1 was used in combination with an NHEJ inhibitor, such as SCR7 or Nu7441, the rate of homologous repair increased significantly to 74% (Zhang et al., 2018). Aksoy et al. (2019) demonstrated that the combination of RS-1 and Nu7441 resulted in HDR rates of 53%, but this was not significantly different from Nu7441 alone (Zhang et al., 2018).

One study compared several small interfering compounds and their effectiveness to improve HDR rates. Boel et al. (2018) showed that no NHEJ inhibitor or HDR enhancer significantly increased HDR rates for somatic integration or germline transmission (Figure 2 and Supplementary Figure 1). In addition to SCR7, Nu7441, and RS-1, KU0060648 and L755507 did not improve HDR success (Boel et al., 2018). NHEJ inhibitors and HDR enhancers did not improve HDR rates for genome modifications using lssDNA templates either (Bai et al., 2020).

When comparing the three studies that used small interfering molecules, two studies co-injected drugs with sgRNA, Cas9, and donor template (Boel et al., 2018; Aksoy et al., 2019). The third study incubated injected embryos in zebrafish embryo media with SCR7, Nu7441, and/or RS-1 (Zhang et al., 2018). Incubation of injected embryos with the small interfering drugs significantly increased HDR rates (74%) (Zhang et al., 2018) when compared to embryos that were injected with drugs (53.7%) (Boel et al., 2018; Aksoy et al., 2019). Although the HDR protocols and target genes varied across all three studies, it should be noted that the highest reported success rates for HDR overall did not use drugs to assist with HDR (Hisano et al., 2015; Bai et al., 2020).

The variability in HDR success observed with different drugs and genes suggests the molecular machinery involved in NHEJ or HDR varies at different loci. This suggests that there are unknown proteins/components of the repair mechanism that are not being targeted. Support for this hypothesis comes from work that explores how different DNA end configurations elicit specific components of the NHEJ pathway to respond (Dorsett et al., 2014; Chang et al., 2016). This would suggest that the effectiveness of HDR drugs is also dependent on the type of break created and whether the break ends are further degraded by exonucleases. While the number of studies with HDR-related drugs is limited, the current results suggest that combinations of small interfering molecules could target core repair proteins in the majority of end joining processes.

### Other Methods

Increasing the time that Cas9/sgRNA can cut and the time the homologous repair pathway has with the target gene before cell division may be another beneficial protocol addition. Incubating injected zebrafish embryos at cooler temperatures for a very short period of time has been shown to increase HDR by 1.5-fold (Aksoy et al., 2019).

### *Tyrosinase* Case Study

*Tyrosinase* provided a unique opportunity to examine four different HDR techniques and protocols on one gene with four success rates (32%, 63.6%, 77%, and 98.5%) (Hisano et al., 2015; Moreno-Mateos et al., 2017; Bai et al., 2020; Wierson et al., 2020). Unlike the majority of genes reviewed, successful modification of *tyr* involves more than DNA topology since *tyr* is not expressed until after 9 hpf (Cas9 active period) and has no neighbors with early expression. To determine what technique/protocol variables may have led to four different HDR rates, we directly compared the experimental designs and results of the four *tyr* studies.

Three studies targeted exon 1 and were within 200 bp of each other (Hisano et al., 2015; Moreno-Mateos et al., 2017; Bai et al., 2020), while the fourth study targeted exon 4 (Wierson et al., 2020). All sgRNAs had cutting efficiencies of 66% or higher. Bai et al. (2020) created a *tyrosinase* mutant (*tyr^25*del*^*) and then rescued their custom mutant using HDR with a success rate of 98.5%. This technique involved Cas9 mRNA, injection into the cell, and lssDNA with ∼135 nt homology arms. Hisano et al. (2015) used Cas9 mRNA, injection into the cell, and a plasmid template with 40 bp arms resulting in 77% HDR success. Wierson et al. (2020) also used Cas9 mRNA, injected into the cell with a plasmid template that contained 24 bp arms, and a somatic success rate of 63.6%. The difference of the latter two techniques is that the plasmid template also required cutting with an sgRNA-Cas9 or endonuclease complex, and this repair inserted a reporter into the target gene. This repair “insertion” is much larger than the 25 bp repair on the *tyr^25*del*^* mutant conducted (Bai et al., 2020). Moreno-Mateos et al. (2017) modified *tyr* using Cpf1 in the cell and ssDNA with asymmetrical homology arms (36 and 71 nt). The homologous repair in this study introduced three stop codons, which is a significantly smaller insertion than any of the other *tyr* protocols. Moreno-Mateos et al. (2017) also modified *tyr* with Cas9 but at a much lower success rate (32%, Cpfl; ∼16%, Cas9).

Based on this comparison, the size of the repair does not seem to affect HDR success rate as there is no trend between insertion size and HDR success rate; the smallest insertion (nine nucleotides/three stop codons) had the lowest HDR rate (32%) (Moreno-Mateos et al., 2017) when compared to the larger insertions into *tyr* (1,500 bp, 63.6%; 39 bp, 77%; 25 bp, 98.5%) (Hisano et al., 2015; Bai et al., 2020; Wierson et al., 2020). Homology arm size also did not follow a trend when comparing the four *tyr* studies. However, the top three *tyr* studies (Hisano et al., 2015; Bai et al., 2020; Wierson et al., 2020) used symmetrical repair templates while the fourth used an asymmetrical template (Moreno-Mateos et al., 2017). This comparison includes a limited number of studies; although template symmetry might be a significant variable for the modification of *tyr*, we cannot definitively conclude that symmetry was the deciding factor for HDR success rates.

## Conclusion

Based on the success of the research reviewed here, we provide protocol suggestions for increasing HDR rates (Table 5). In addition, the ratio of endonuclease injected into the embryo should be more than either sgRNA or donor template, although there are successful experiments that did not use this combination.

**TABLE 5:** Recommendations for variable steps to maximize homology-directed repair (HDR) success in zebrafish.

inject into the 1 or 2-cell cytoplasm endonuclease mRNA ssDNA repair template symmetrical or asymmetrical 18-400 nucleotides for either arm interfering drugs may have a positive effect incubate injected embryos in drugs combination drugs recommended

*See text for discussion. We compared all of the protocols and identified all of the steps where there was variability and analyzed those variables to determine what gave the best HDR rate.*

It would be highly informative to study HDR success rates comparing these variables for zebrafish genome HDR across a subset of genes. Such a study would be an enormous undertaking but would create a foundation for precise modification of the zebrafish genome.

## Author Contributions

KP and JD contributed to the conceptualization, writing, and editing or formatting. KP contributed to data analysis. JD acquired funding. Both authors contributed to the article and approved the submitted version.

## Conflict of Interest

The authors declare that the research was conducted in the absence of any commercial or financial relationships that could be construed as a potential conflict of interest.

## Footnotes

1 In the literature, homologous directed repair (HDR) is also referred to as: homologous repair, homology recombination repair, knock-ins, homology-driven recombination, ssODN-mediated-homology-directed repair, ssODN-mediated gene repair and precise genome modification. In this review, we will refer to all of these techniques as homologous directed repair (HDR).
