# Supplementary material for: Homology-Directed Repair in Zebrafish: Witchcraft and Wizardry?
Source: Front Mol Biosci. 2020 Dec 7;7:595474. doi: 10.3389/fmolb.2020.595474 (PMC7793982; doi:10.3389/fmolb.2020.595474)
Supplement: Supplementary file 1 [file Table_1.DOCX]

Supplementary Material
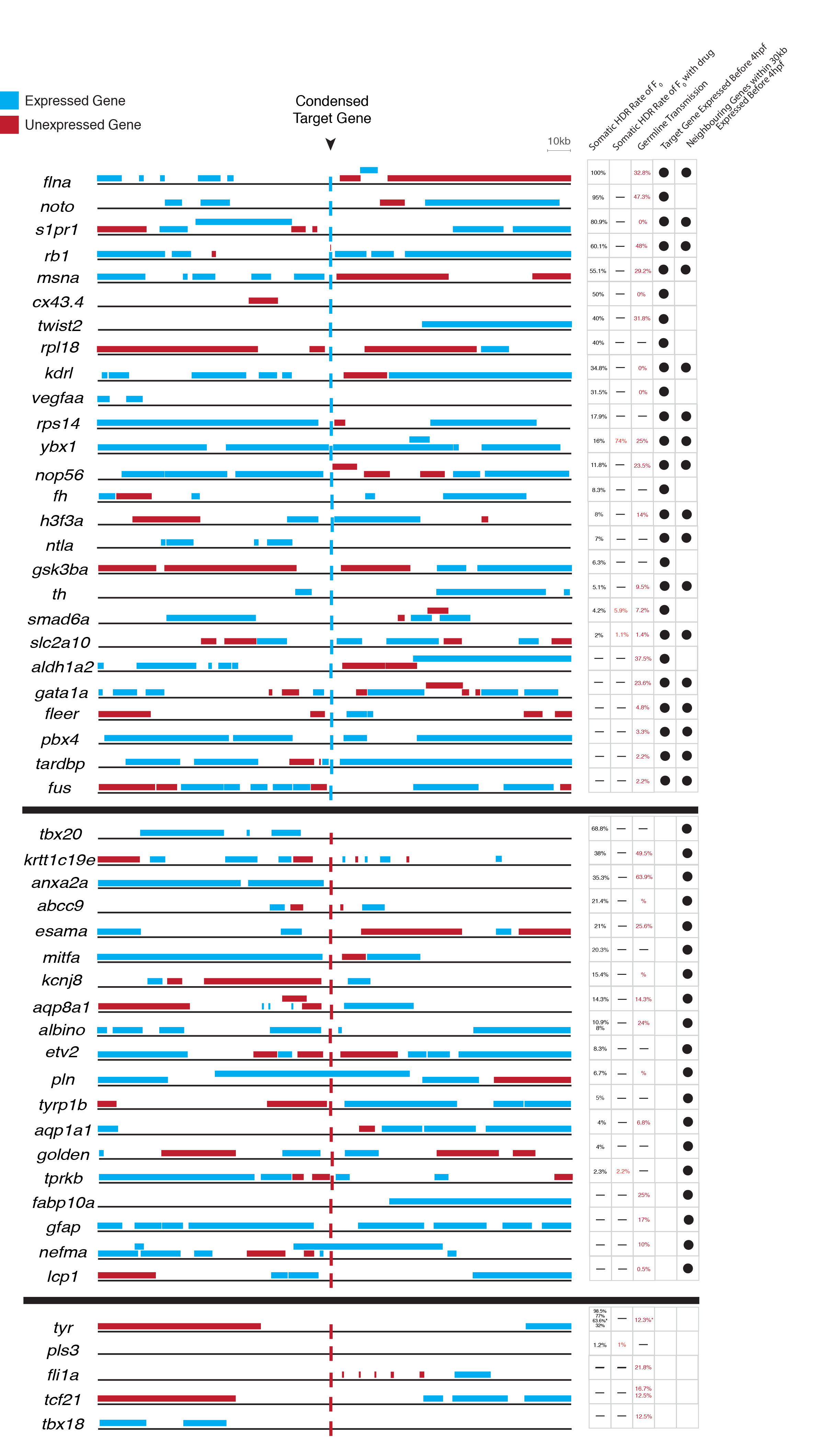


**Supplementary Figure 1.** Expression status of neighbouring genes and 50 target genes successfully modified using HDR. Genes are initially sorted according to target and neighbouring gene expression (black dots). Genes are further ranked in descending somatic HDR rates followed by descending germline transmission rates when no somatic rate was provided. Target genes are condensed and centered on the local chromosome region (thin black line) with their names displayed on the left. Neighbouring genes are represented by colored bars with genes expressed before 4 hpf shown in blue and genes expressed after 4 hpf shown in red; target genes and their expression are represented with the same color designation. Target and neighbouring gene expression are simplified in the columns with a black dot indicating the period of expression. Spatial arrangement of the genomic region is displayed by alternating grey and white bars with each representing 10kb. A percent sign without a rate represents a study where success was mentioned but no rate was provided or could be determined. Dashes in the rate column indicate studies that did not mention a success in that tissue. Neighbouring genes with no expression data are not represented. Asterisk indicates HDR rates from the same study.
